# Neuroimaging predictors of treatment response in anxiety disorders

**DOI:** 10.1186/2045-5380-3-15

**Published:** 2013-08-02

**Authors:** Lisa M Shin, F Caroline Davis, Michael B VanElzakker, Mary K Dahlgren, Stacey J Dubois

**Affiliations:** 1 Department of Psychology, Tufts University, Medford, MA, USA; 2 Department of Psychiatry, Massachusetts General Hospital, Charlestown, MA, USA

**Keywords:** Social anxiety disorder, Obsessive-compulsive disorder, Posttraumatic stress disorder, Generalized anxiety disorder, Amygdala, Medial prefrontal cortex, Anterior cingulate cortex, Orbitofrontal cortex, fMRI, PET

## Abstract

Although several psychological and pharmacological treatment options are available for anxiety disorders, not all patients respond well to each option. Furthermore, given the relatively long duration of adequate treatment trials, finding a good treatment fit can take many months or longer. Thus, both clinicians and patients would benefit from the identification of objective pre-treatment measures that predict which patients will best respond to a given treatment. Recent studies have begun to use biological measures to help predict symptomatic change after treatment in anxiety disorders. In this review, we summarize studies that have used structural and functional neuroimaging measures to predict treatment response in obsessive-compulsive disorder (OCD), posttraumatic stress disorder (PTSD), generalized anxiety disorder (GAD), and social anxiety disorder (SAD). We note the limitations of the current studies and offer suggestions for future research. Although the literature is currently small, we conclude that pre-treatment neuroimaging measures do appear to predict treatment response in anxiety disorders, and future research will be needed to determine the relative predictive power of neuroimaging measures as compared to clinical and demographic measures.

## Review

Anxiety disorders are highly prevalent [1] and are associated with occupational disability and increased family burden [2-4]. Although psychological and pharmacological treatments are available, they are not always effective. For example, a recent naturalistic study of obsessive-compulsive disorder (OCD) revealed that only approximately two-thirds of individuals taking serotonin reuptake inhibitors (SRIs) considered their symptoms very much or much improved [5]. The percentage of individuals responding to pharmacotherapy appears to be even lower in posttraumatic stress disorder (PTSD) [6]. Although cognitive-behavioral therapies are moderately effective in the treatment of anxiety disorders, there appears to be room for improvement [7-9], perhaps especially in the case of panic disorder and generalized anxiety disorder (GAD) [8].

Given the variability in treatment response, it would be beneficial to determine whether measures obtained before treatment could help clinicians predict symptomatic change in response to a given treatment. Several clinical measures have been useful in this regard. For example, greater pre-treatment symptom severity has been associated with less favorable response to treatment in OCD [10], PTSD [11,12], social anxiety disorder (SAD) [13,14], and in youth with anxiety disorders [15]. Other clinical variables have predicted a less favorable response to treatment, such as the presence of hoarding obsessions and compulsions [16] and comorbid personality disorders in OCD [17] and greater depression and avoidant personality traits in SAD [18].

Recent studies have begun to use biological measures to predict symptomatic change after treatment in anxiety disorders (e.g., [19-22]). Such measures are more objective and arguably more proximal to the neurobiological substrates of these disorders as compared to symptom severity measures. Structural and functional neuroimaging techniques yield such biological measures (e.g., regional cerebral metabolic rate for glucose [rCMRglu] in the anterior cingulate cortex [ACC]) that can be examined for possible associations with treatment response. Here, we will review the findings of longitudinal studies that used pre-treatment structural and functional neuroimaging measures to predict treatment-related symptomatic change in anxiety disorders.

## Methodological considerations

The studies reviewed below implemented a variety of different imaging techniques and data analytic approaches. Early studies tended to use positron emission tomography (PET) and fluorodeoxyglucose (FDG) to measure rCMRglu while patients rested with eyes open. Although such resting state studies are relatively easy to administer because they require no explicit cognitive task, they are also limited by a lack of control over the mental processes in which subjects engage during the radiotracer uptake period. This can lead to greater variability in the data, perhaps increasing the risk of type II errors (depending on the kinds of analyses used), as well as some difficulty interpreting the meaning of the rCMRglu findings. In general, PET-FDG imaging is also limited by relatively poor temporal resolution as rCMRglu data are typically averaged across long periods of time (~30-45 minutes).

Many older PET-FDG studies also used region-of-interest (ROI) based analyses in which researchers draw boundaries around brain regions based on structural anatomy, “extract” functional imaging data from those drawn regions, and submit the extracted data to external statistical software. One disadvantage of this technique is that it involves averaging functional imaging data across very large brain structures, increasing the risk of type II error if only a small portion of that large brain structure is actually important in the prediction of treatment response. More recent neuroimaging studies employ voxelwise analyses that can assess the relationship between brain activation (or gray matter volume) and treatment response at every voxel (e.g., 3 mm × 3 mm × 3 mm cube) in the brain. However, in order to complete such analyses, one must “normalize” or morph the functional (and/or structural) brain images to a standard brain template, which introduces some amount of error. In addition, voxelwise analyses involve conducting thousands of analyses (one per voxel) across the brain, so correction for multiple comparisons must be applied.

More recent studies have implemented functional magnetic resonance imaging (fMRI) to measure brain activation in response to cognitive and/or affective tasks. These types of studies afford more control over the cognitive state of the participant during scanning and, if well designed, allow for a more clear interpretation of the meaning of the imaging findings.

The methodological considerations described above apply to most functional neuroimaging studies, not just those assessing the prediction of treatment response. With regard to the latter specifically, researchers have used two different data analytic approaches. The first approach involves comparing treatment responders to non-responders on a pre-treatment neuroimaging measure (e.g., amygdala activation). For example, if treatment responders as a group were found to have lower pre-treatment amygdala activation than non-responders, then lower amygdala activation would be considered to be predictive of a better response to treatment. This type of between-group analysis typically involves analysis of variance (ANOVA) or analysis of covariance (ANCOVA), and requires (1) a very clear and well-accepted definition of treatment response and (2) sufficiently large numbers of participants per group (responders and non-responders). Because the relative size of these post-treatment groups cannot be completely planned a priori, these studies should begin with a very large number of participants.

The second approach is to run a correlation (or a regression) between a continuous pre-treatment neuroimaging measure and a continuous measure of symptomatic improvement (e.g., symptom change score, percent improvement, or clinical global impression (CGI) improvement score). This type of analysis takes advantage of the inherent variability in both the imaging and treatment response data and is therefore likely to be more powerful than the between-group approach. Regression analyses also obviate the need to form responder and non-responder groups and permit the investigation of multiple pre-treatment imaging (and non-imaging) variables in the prediction of treatment response. For example, baseline symptom severity and comorbidity measures (either categorical or continuous) can be entered into the regression along with pre-treatment imaging measures to predict symptom change scores. Several studies reviewed below included both types of approaches.

In the following text, we review studies that have assessed whether pre-treatment structural or functional neuroimaging measures can predict treatment response in OCD, PTSD, GAD, and SAD (See Table 1). (We were able to find no such studies of panic disorder or specific phobia). We did not include studies that assessed the *change* in neuroimaging measures with treatment as those studies address a different question.

**Table 1:** Summary of Neuroimaging studies predicting treatment response in anxiety disorders

**Article**	**Disorder**	**Imaging**	**Treatment type**	**Sample sizes**	**Outcome measure**	**Findings**
Swedo et al. [32]	OCD	PET-FDG: resting state	Clomipramine (dose and duration not specified)	OCD: 18 Healthy: 18	OCR Responders: ≥ 40% reduction in OCR (11 R, 6 NR)	Pre-treatment rCMRglu in the right ACC and right OFC was lower in clomipramine R vs. NR.
Saxena et al. [33]	OCD	PET-FDG: resting state	Paroxetine (8-12 weeks; 40 mg/d max)	OCD: 20	YBOCS and CGI Responders: ≥ 25% reduction in YBOCS and CGI of much improved or very much improved (11 R, 9 NR)	Lower pre-treatment rCMRglu in bilateral OFC predicted better response to paroxetine.
Saxena et al. [34]	OCD, MDD, OCD + MDD	PET-FDG: resting state	Paroxetine (8-12 weeks; 30-60 mg/d)	OCD: 27 MDD: 27 OCD + MDD: 17	YBOCS, HAM-D	Greater pre-treatment rCMRglu in the caudate predicted greater improvement in OCD symptoms in the OCD groups. Lower rCMRglu in the amygdala predicted more improvement in MDD symptoms in MDD group and in all Ss combined. Greater pre-treatment rCMRglu in the medial frontal gyrus predicted improvement in MDD symptoms in all Ss.
Hendler et al. [35]	OCD	SPECT: symptom provocation vs. relax	Sertraline (6 months; 200 mg/d max)	OCD: 26	YBOCS Responders: ≥ 30% reduction in YBOCS (13 R, 13 NR)	R had lower pre-treatment perfusion during symptom provocation in dorsal/caudal ACC and higher perfusion in right caudate vs. NR.
Rauch et al. [36]	OCD	PET-015: symptom provocation vs. neutral	Fluvoxamine (12 weeks; 300 mg/d max)	OCD: 9	YBOCS	Lower rCBF in OFC and higher rCBF in PCC predicted better response.
Sanematsu et al. [37]	OCD	fMRI: symptom provocation vs. neutral	Fluvoxamine (12 weeks; 200 mg/d max)	OCD: 17	YBOCS	Pretreatment activation of right cerebellum and left superior temporal gyrus was positively correlated with YBOCS improvement.
Ho Pian et al. [38]	OCD	SPECT: resting state	Fluvoxamine (12 weeks; 300 mg/d max)	OCD: 15	YBOCS Responders: ≥25% reduction in YBOCS (7 R, 8 NR)	Pre-treatment cerebellar and whole brain rCBF was significantly higher in R vs. NR.
Buchsbaum et al. [39]	OCD	PET-FDG: resting state	Risperidone or placebo augmentation (8 weeks; 3 mg/d max)	OCD: 15 Risperidone: 9 Placebo: 6	YBOCS Responders: ≥ 25% reduction in YBOCS and/or CGI Improvement rating of very much improved or much improved (4 R, 5 NR)	Pre-treatment rCMRglu was lower in the striatum and higher in the ventral ACC in R vs. NR.
Brody et al. [40]	OCD	PET-FDG: resting state	Fluoxetine (10 weeks; 60 mg/d) or group BT (10 weeks)	OCD: 27 Fluoxetine: 9 CBT: 18	YBOCS	Greater pre-treatment rCMRglu in the left OFC was associated with a better response to BT. In this same region, lower rCMRglu was associated with better response to fluoxetine.
Hoexter et al. [41]	OCD	mMRI	Fluoxetine (12 weeks; 80 mg/d max) or group CBT (12 weekly sessions)	OCD: 29 Fluoxetine: 14 CBT: 15	YBOCS	Lower pre-treatment gray matter density in ventrolateral prefrontal cortex predicted better response to fluoxetine. Greater gray matter density in subgenual ACC predicted better response to CBT.
Rauch et al. [44]	OCD	PET-FDG: resting state	Anterior cingulotomy	OCD: 11	YBOCS	Greater pre-operative rCMRglu in posterior cingulate predicted greater improvement.
Van Laere et al. [45]	OCD	PET-FDG: resting state	Stimulation of anterior capsule	OCD: 6 Controls: 20	YBOCS	Greater pre-operative rCMRglu in the subgenual ACC predicted greater improvement.
Bryant et al. [68]	PTSD	mMRI	CBT (8 weekly sessions)	PTSD: 13 TENP: 13 Healthy: 13	CAPS Responders: no longer met diagnostic criteria (7 R, 6 NR)	Greater pre-treatment gray matter density in the rACC predicted greater improvement.
Bryant et al. [19]	PTSD	fMRI: masked fearful vs. neutral faces	CBT (8 weekly sessions)	PTSD: 14 Healthy: 14	CAPS Responders: ≥ 50% reduction in CAPS (7 R, 7 NR)	Lower pre-treatment amygdala and rACC activation predicted greater improvement.
Nardo et al. [69]	PTSD	mMRI	EMDR (5 sessions)	PTSD: 21 TENP: 22	Responders: no longer met diagnostic criteria (10 R, 5 NR)	R had greater gray matter density in the insula, amygdala/parahippocampal gyrus, posterior cingulate, and middle, precentral, and dorsal medial frontal gyri.
Whalen et al. [75]	GAD	fMRI: fearful vs. neutral/happy faces	Venlafaxine (8 weeks; 225 mg/d max)	GAD: 15 Healthy: 15	HAM-A	Lower pre-treatment amygdala activation and greater rACC activation predicted greater improvement in anxiety.
Nitschke et al. [73]	GAD	fMRI: anticipation of aversive vs. neutral images	Venlafaxine (8 weeks; 225 mg/d max)	GAD: 14 Healthy: 12	HAM-A and Penn State Worry Questionnaire	Greater pre-treatment rACC activation predicted greater improvement in anxiety.
McClure et al. [76]	GAD	fMRI: fearful vs. happy faces	Fluoxetine (8 weeks; 40 mg/d max) or CBT (8 weekly sessions)	GAD: 12 Fluoxetine: 5 CBT: 7	CGI	Greater pre-treatment amygdala activation predicted greater improvement.
Evans et al. [87]	SAD	PET-FDG: resting state	Tiagabine (6 weeks; 16 mg/d max)	SAD: 12 Healthy: 10	LSAS Responders: ≥ 50% reduction in LSAS scores (7 R, 5 NR)	Voxelwise correlations were not significant. Pre-treatment rCMRglu was lower in subcallosal ACC in R compared to healthy controls.
Doehrmann et al. [13]	SAD	fMRI: 1-back task, angry vs. neutral faces	CBT (12 weekly sessions)	SAD: 39	LSAS	Greater pre-treatment activation in dorsal and ventral occipitotemporal cortex predicted greater improvement.

*ACC* anterior cingulate cortex, *BT* behavioral therapy, *CAPS* Clinician Administered PTSD Scale, *CBT* cognitive-behavioral therapy, *CGI* Clinical Global Impression scale, *EMDR* eye movement desensitization and reprocessing, *fMRI* functional magnetic resonance imaging, *GAD* generalized anxiety disorder, *HAM-A* Hamilton Rating Scale for Anxiety, *HAM-D* Hamilton Rating Scale for Depression, *LSAS* Liebowitz Social Anxiety Scale, *MDD* major depressive disorder, *mg/d* milligrams per day, *mMRI* morphometric magnetic resonance imaging, *NR* non-responders, *OCD* obsessive-compulsive disorder, *OCR* Obsessive Compulsive Rating scale, *OFC* orbitofrontal cortex, *PET-FDG* positron emission tomography with fluorodeoxyglucose, *PET-015* positron emission tomography with oxygen-15, *PTSD* posttraumatic stress disorder, *R* responders, *rACC* rostral anterior cingulate cortex, *rCBF* regional cerebral blood flow, *rCMRglu* regional cerebral metabolic rate for glucose, *SAD* social anxiety disorder, *SPECT* single photon emission computed tomography, *Ss* subjects, *TENP* trauma-exposed non-PTSD, *YBOCS* Yale-Brown Obsessive-Compulsive Scale.

### Obsessive-compulsive disorder (OCD)

In contrast to other anxiety disorders, OCD appears to be marked by structural and functional abnormalities in thalamo-cortico-striatal loops. One neurocircuitry model of OCD [23,24] posits that the striatum (caudate nucleus) functions abnormally, leading to inefficient gating in the thalamus. This leads to hyperactivity in the orbitofrontal cortex and the ACC, which may mediate intrusive thoughts and anxiety, respectively. Compulsions recruit the striatum to achieve thalamic gating, thereby neutralizing the obsessions and reducing anxiety. Indeed, several resting state and symptom provocation functional neuroimaging studies have revealed greater activation of the caudate, thalamus, orbitofrontal cortex, and/or ACC in OCD (e.g., [25-28], although the direction of the abnormalities is not entirely uniform across studies). Pre-treatment functional abnormalities in these structures appear to resolve with successful treatment (e.g., [29-31]). Several studies have examined pre-treatment neuroimaging predictors of response to medication and/or behavioral therapy (BT) or cognitive-behavioral therapy (CBT) in OCD.

#### Medication

In a very early PET study of OCD, Swedo and colleagues [32] found that pre-treatment rCMRglu in the right ACC and right orbitofrontal cortex was lower in clomipramine responders vs. non-responders. They also reported a positive correlation between pre-treatment symptom severity and pre-treatment rCMRglu in the orbitofrontal cortex, suggesting that treatment response is likely better in those participants with less severe symptoms. Saxena and colleagues [33] reported similar findings in a PET study of paroxetine. Specifically, lower pre-treatment rCMRglu in bilateral orbitofrontal cortex was associated with better improvement.

Another PET study of response to paroxetine [34] examined the more complicated question of whether pre-treatment rCMRglu could differentially predict improvement in OCD symptoms vs. depression symptoms in patients with OCD alone, comorbid OCD and MDD, and MDD alone. This study was unique in that it utilized both ROI-based and voxelwise analyses, the results of which were partially but not completely convergent. ROI-based analyses showed that greater pre-treatment rCMRglu in the caudate predicted greater improvement in OCD symptoms in the groups with OCD, but did not predict improvement in depression symptoms in any group. These findings were not replicated in the voxelwise analyses. ROI-based analyses also showed that lower pre-treatment rCMRglu in the amygdala predicted greater improvement in depression symptoms in the MDD group and in all subjects combined. Voxelwise analyses confirmed this finding in all subjects and further showed that greater pre-treatment rCMRglu in the medial frontal gyrus (just anterior to the rostral ACC [rACC]) predicted improvement in depression symptoms in all subjects regardless of diagnostic group. These findings are important because they suggest that (1) rCMRglu predictors of improvement differ for different types of symptoms even in the same subjects, and (2) rCMRglu predictors of improvement can cut across diagnostic lines.

Hendler et al. [35] used single photon emission computed tomography (SPECT) to determine whether pre-treatment regional cerebral perfusion during symptom provocation could predict response to sertraline in individuals with OCD. Treatment responders showed lower pre-treatment perfusion in dorsal/caudal ACC and higher pre-treatment perfusion in right caudate compared to non-responders. These findings were not observed when the SPECT measures were obtained during the relaxed (unprovoked) state. Thus, these findings suggest that functional imaging-based measures obtained in one state (e.g., symptomatic) may predict treatment response in only that state and not others.

In contrast, Rauch and colleagues [36] found that state of the participants did not affect the prediction of response to fluvoxamine in OCD. In a PET study, these authors found that pre-treatment regional cerebral blood flow (rCBF) measured in neutral and symptomatic states similarly predicted treatment response: lower pre-treatment rCBF in orbitofrontal cortex and greater pre-treatment rCBF in the posterior cingulate cortex predicted better response to fluvoxamine.

Unlike previous studies of treatment response in OCD, Sanematsu and colleagues [37] used fMRI to examine neural predictors of improvement. Functional MRI data were collected while participants generated either words related to their OCD symptoms or control words (relating to flowers and vegetables). Greater pre-treatment activation in the right cerebellum and left superior temporal gyrus was associated with better response to fluvoxamine. Similarly, Ho Pian and colleagues [38] found that pre-treatment activity (as measured by 99mTc-HMPAO tracer uptake) in the cerebellum was greater in responders vs. non-responders to fluvoxamine.

In a study of OCD patients who were non-responders to SRIs, Buchsbaum and colleagues [39] found that responders to risperidone augmentation had lower pre-treatment rCMRglu in the striatum and higher pre-treatment rCMRglu in the ventral ACC. These findings differ from those of previous studies of OCD most likely because of the nature of both the patient group (SRI non-responders) and the treatment (antipsychotic augmentation).

#### Medication and BT/CBT

In what appears to be the first study to examine the neuroimaging predictors of response to two different treatments for OCD, Brody et al. [40] studied pre-treatment rCMRglu in patient groups who chose to receive either BT or fluoxetine. They found that greater pre-treatment rCMRglu in the left orbitofrontal cortex was significantly associated with greater symptomatic improvement after BT. Interestingly, within this same ROI, lower pre-treatment rCMRglu was associated with greater improvement after treatment with fluoxetine.

In a similar study, Hoexter and colleagues [41] examined structural imaging predictors of treatment response in treatment-naïve patients randomly assigned to receive either fluoxetine or group CBT. Using voxel-based morphometry, they found that lower pre-treatment gray matter density in ventrolateral frontal cortex predicted greater improvement in OCD symptoms after treatment with fluoxetine. In contrast, greater pre-treatment gray matter density in subgenual ACC predicted greater improvement in OCD symptoms after CBT. When the two treatment groups were combined, there were no significant effects. Given that CBT involves extinction-like processes and that ventral medial prefrontal cortex (mPFC) is critical for the retention of extinction memory [42,43], it makes sense that patients with greater pre-treatment gray matter volume in ventral mPFC would show greater symptomatic improvement with CBT. Along with Brody et al. [40], this study suggests different imaging predictors of response to medication vs. CBT in OCD.

#### Neurosurgery

Identifying reliable and valid predictors of treatment response is even more critical when the treatment is invasive and associated with elevated risk, such as the case with neurosurgery. Rauch and colleagues [44] evaluated the PET predictors of response to anterior cingulotomy and found that relatively greater pre-treatment rCMRglu in the posterior cingulate cortex predicted greater improvement in OCD symptom severity after surgery. These findings were consistent with those of Rauch et al. [36] in the prediction of response to fluvoxamine. In a study of anterior capsule stimulation, Van Laere and colleagues [45] found that greater pre-operative rCMRglu in the subgenual ACC predicted greater improvement in OCD symptom severity.

In summary, lower pre-treatment activity in the orbitofrontal cortex and greater activity in the caudate and posterior cingulate cortex predict a more favorable response to SRIs or neurosurgery in OCD. In contrast, greater pre-treatment activity or gray matter volume in ventral mPFC appears to predict better response to BT/CBT. This latter finding has also been reported in PTSD.

### Posttraumatic stress disorder (PTSD)

Some neurocircuitry models [46,47] posit that the amygdala is hyperresponsive in PTSD, perhaps accounting for hypervigilance, increased arousal, and the persistence of trauma-related memories. In addition, the mPFC (including the rACC) is thought to be hyporesponsive, with diminished inhibition over the amygdala, and this may underlie extinction memory deficits and difficulty ignoring trauma-related reminders. Neuroimaging studies of PTSD have generally reported increased activation in the amygdala, insular cortex, and dorsal anterior cingulate cortex (dACC), and decreased activation in the mPFC [48-51]. In addition, structural neuroimaging studies have reported decreased volume or gray matter density in the amygdala, mPFC, and hippocampus in this disorder (e.g., [52-55]). Furthermore, PTSD symptom severity measures are often correlated positively with amygdala activation (e.g. [56,57]) and negatively with mPFC activation (e.g., [58-61]). A few studies have suggested that amygdala activation decreases and mPFC activation increases with successful treatment [62-65].

The use of exposure-based techniques to treat PTSD is well supported in the literature (e.g., [66,67]) and most studies that have examined neuroimaging predictors of treatment response have implemented such techniques. Bryant et al. [68] used structural MRI and voxel-based morphometry to determine whether pre-treatment gray matter density predicted response to CBT in participants with PTSD. Correlational analyses revealed that pre-treatment gray matter density in the rACC was positively correlated with symptomatic improvement, even after controlling for depression and baseline PTSD symptom severity. Indeed, treatment responders, trauma-exposed comparison subjects without PTSD, and healthy comparison subjects had higher pre-treatment gray matter density in the rACC than did treatment non-responders.

In an fMRI study, Bryant and colleagues [19] presented backwardly-masked fearful vs. neutral facial expressions to participants with PTSD before they completed eight sessions of CBT. Treatment response was assessed 6 months after treatment completion. Voxelwise correlational analyses revealed that lower pre-treatment activation in the amygdala and rACC predicted better symptomatic improvement after CBT. Between-group comparisons (non-responders vs. responders) confirmed these findings. Of note, the finding in the rACC was opposite of prediction.

In a voxel-based morphometry study of railway workers with PTSD, Nardo et al. [69] found that compared to non-responders, responders to eye-movement desensitization and reprocessing (EMDR) treatment had greater pre-treatment gray matter density in large territories of the brain including the insula, amygdala/parahippocampal gyrus, posterior cingulate, and middle, precentral, and dorsal medial frontal gyri.

In summary, pre-treatment neuroimaging measures of the amygdala and mPFC (specifically, the rACC) predict response to BT/CBT in PTSD, although additional studies are needed to specify the direction of the findings. Interestingly, mPFC activation also appears to predict treatment response in GAD, as shown below.

### Generalized anxiety disorder (GAD)

Although relatively few functional neuroimaging studies of GAD exist in the literature, there has been some support for increased activation in the amygdala and mPFC in GAD relative to comparison groups ([70-73], but see also [74,75]). One study reported a positive correlation between amygdala activation and GAD symptom severity [72]. Three fMRI studies have examined neuroimaging predictors of treatment response in GAD.

Whalen and colleagues [75] found that pre-treatment activation in the amygdala in response to fearful (vs. neutral or happy) facial expressions was negatively correlated with symptomatic change after treatment with venlafaxine in GAD. Specifically, relatively lower pre-treatment amygdala responses were associated with relatively greater improvement. Interestingly, the opposite relationship was found in the rACC: the greater the pre-treatment rACC activation, the greater the improvement.

Using a different fMRI paradigm, Nitschke and colleagues [73] found that pre-treatment rACC activation during the anticipation of aversive and neutral pictures was inversely correlated with post-treatment symptoms of anxiety and worry (controlling for pre-treatment symptoms) in participants treated with venlafaxine. Thus, consistent with the findings of Whalen et al. [75], relatively greater pre-treatment rACC activation predicted relatively greater improvement. The authors found no significant association between pre-treatment amygdala responses and symptomatic change.

In contrast to the findings of Whalen et al. [75], McClure and colleagues [76] examined amygdala activation in twelve adolescents with anxiety disorders (nine had primary GAD) before treatment with either CBT or fluoxetine. Participants viewed fearful and happy faces in the scanner and responded to the question “How afraid are you?” Pre-treatment amygdala activation to fearful vs. happy faces was inversely associated with post-treatment symptom severity, even after controlling for pre-treatment symptom severity. That is, the greater the pretreatment amygdala activation, the better the treatment response. The discrepancy between this finding and that of Whalen et al. [75] could have been due to the younger age and/or the diagnostic heterogeneity in the McClure et al. sample (not all of the participants had GAD).

In summary, the amygdala and mPFC appear to predict treatment response in GAD, although the direction of the findings remains to be confirmed. Two of the three studies, however, suggest that greater pre-treatment mPFC (specifically, rACC) activation is related to better response to venlafaxine.

### Social anxiety disorder (SAD)

Functional neuroimaging studies of SAD have revealed exaggerated amygdala activation (e.g. [77-79]), which is positively correlated with symptom severity (e.g., [80,81]) and appears to decrease after successful treatment [82,83]. Insular cortex also appears to be hyperresponsive in SAD relative to comparison groups (e.g., [84-86]). The direction of functional abnormalities in the mPFC in this disorder is less clear, with some studies reporting increased activation and others reporting decreased activation [51]. Only two studies have used neuroimaging measures to predict treatment response in SAD.

In a PET-FDG study, Evans and colleagues [87] found no significant voxelwise correlations between pre-treatment rCMRglu in their regions of interest (which included mPFC/ACC, amygdala, hippocampus, parahippocampal gyrus, insula) and symptomatic improvement after treatment with tiagabine. However, categorical group comparisons revealed that treatment responders had lower pre-treatment rCMRglu in subcallosal gyrus compared to healthy control subjects. Furthermore, rCMRglu within this region was inversely correlated with symptomatic improvement across all treated participants (i.e., relatively lower pre-treatment rCMRglu was associated with relatively greater improvement). Treatment non-responders had lower baseline rCMRglu in the dACC than control subjects, but rCMRglu in the dACC was not correlated with symptomatic improvement. It should be noted that the type of categorical group comparisons used in this study (i.e., responder vs. healthy controls and non-responder vs. healthy controls) differed from that of other studies (i.e., responder vs. non-responder) reviewed herein. This data analytic difference could very well account for discrepancies between the findings of this and other studies.

Doehrmann and colleagues [13] used fMRI to examine pre-treatment predictors of response to CBT in a relatively large sample (n = 39) of individuals with SAD. During scanning, participants performed a 1-back task on sets of visual stimuli that contained facial expressions (angry and neutral) and scenes without people (emotional and neutral). Correlational analyses showed that higher pre-treatment activation in dorsal and ventral regions of occipitotemporal cortex in response to angry vs. neutral faces was associated with greater symptomatic improvement. In multiple regression analyses predicting symptomatic improvement, the variance explained by the model was significantly greater when activation data from the dorsal and ventral occipitotemporal cortex were added over and above clinical measures such as pre-treatment symptom severity. Unlike previous findings reported in PTSD and GAD [19,75], amygdala activation did not significantly predict symptomatic improvement. This study appears to be the first to demonstrate the added benefit of neuroimaging measures over and above clinical measures in predicting treatment response in anxiety disorders. However, occipitotemporal cortex is not typically among the brain regions found to function abnormally in anxiety disorders.

In summary, only two studies have examined neuroimaging predictors of treatment response in SAD, and those two studies differed quite dramatically in terms of imaging methods, sample sizes, treatment type, and findings. This variability and paucity of data prevent us from drawing general conclusions about the neuroimaging predictors of treatment response in SAD at the present time.

## Conclusions

In summary, although this literature is currently small, pre-treatment neuroimaging measures do appear to predict treatment response in anxiety disorders. Despite the variability in the findings within disorder categories, some consistent patterns have emerged. For example, in OCD, lower pre-treatment activity in the orbitofrontal cortex and greater activity in the caudate and posterior cingulate cortex appear to predict a more favorable response to treatment with SRIs or neurosurgery. In OCD, PTSD, and GAD, greater pre-treatment activity or gray matter density in the mPFC predicts better response to treatment (BT/CBT in OCD and PTSD, and venlafaxine in GAD). In PTSD and GAD, lower pre-treatment amygdala activation predicts a more favorable response to treatment (with CBT in PTSD and venlafaxine in GAD).

Consistent with neurocircuitry models of OCD (e.g., [23]), caudate activity predicted treatment response only in OCD and not in the other anxiety disorders examined herein. In contrast, mPFC neuroimaging measures predicted treatment response in all of the anxiety disorders examined, except for SAD (of which there are too few studies).

Confidence in these findings is tempered, however, by several limitations and caveats. First, as mentioned previously, the present literature is small and includes studies with relatively small numbers of subjects. Second, data analytic methods varied across studies and this alone could account for much of the variability in findings. Third, some studies did not appear to control for pre-treatment symptom severity, leaving open the question of whether neuroimaging measures are merely acting as proxies for baseline symptom severity in the prediction of treatment response. In order for neuroimaging measures to be practical in the prediction of treatment response, they would need to predict treatment response over and above the common clinical predictors, which are easier and less expensive to obtain. The findings of Doehrmann and colleagues [13] are especially encouraging in this regard. In our view, future studies should include regression analyses in which pre-treatment neuroimaging measures, baseline symptom severity, and other clinical (e.g., severity of comorbid disorders), psychophysiologic (e.g., heart rate responses), cognitive (e.g., response times on an interference task) and/or genotype measures are entered as predictors of treatment response (also measured continuously). These types of analyses benefit from the variability inherent in both the predictor and treatment response data and are potentially more powerful than comparisons between responders and non-responders. Furthermore, the most accurate predictions of treatment response will likely result from the consideration of multiple types of predictors.

Fourth, because most studies did not use placebo control groups, it is possible that neuroimaging measures predict improvement in general rather than treatment response per se (as improvement could be due to expectancy effects). This may be less of a problem in studies that compared responders to non-responders if both groups had similar expectancies.

Fifth, most of the studies reviewed above predicted response to one type of treatment. While this is a necessary first step, future studies will need to include more than one treatment type [40,41] in order to truly inform clinical decision-making.

Sixth, future studies will need to assess whether neuroimaging predictors of treatment response are specific to diagnostic categories. Furthermore, given the substantial comorbidity between anxiety disorders and depression, whether neuroimaging predictors of treatment response differ between these two diagnostic categories (and between comorbid and non-comorbid groups) will also need to be evaluated. A brief examination of the separate literatures reveals that some neuroimaging predictors of treatment response appear to be similar between anxiety disorders and depression (amygdala and rACC activation) [88,89], but only one research group has actually examined these different diagnostic groups (OCD, depression, and comorbid OCD/depression) in the same study [34].

Finally, arguably the most important big-picture question for future research to consider is whether the information gleaned from group studies (such as the ones described herein) can eventually be applied to clinical decision making in individual patients. Group studies could yield, for example, logistical regression equations or more complex classification schemes in which future individual patient data could be entered to predict a dichotomous treatment response outcome. This approach has been used in other areas of clinical research [90,91], but not yet in the current one. In the near future, machine learning data analytic techniques, which detect patterns in complex datasets in a bottom-up fashion, could potentially help discriminate between treatment responders and non-responders. Indeed, such techniques are already being used on neuroimaging data to discriminate between individuals with and without psychiatric disorder [92-95]. More generally, studies of neuroimaging predictors of treatment response could contribute to future clinical decision making in individual patients by identifying both (1) impaired neural circuits that could become targets of more directed (or adjunctive) treatments, and (2) other related biomarkers (e.g., skin conductance responses to conditioned stimuli or heart rate responses to loud tones) that may equally predict treatment response and are less expensive and/or more widely available than neuroimaging.

## Abbreviations

ACC: Anterior cingulate cortex; BT: Behavioral therapy; CBT: Cognitive-behavioral therapy; dACC: Dorsal anterior cingulate cortex; fMRI: Functional magnetic resonance imaging; GAD: Generalized anxiety disorder; mPFC: Medial prefrontal cortex; OCD: Obsessive-compulsive disorder; PET: Positron emission tomography; PTSD: Posttraumatic stress disorder; rACC: Rostral anterior cingulate cortex; rCMRglu: Regional cerebral metabolic rate for glucose; rCBF: Regional cerebral blood flow; ROI: Region of interest; SPECT: Single photon emission computed tomography; SRI: Serotonin reuptake inhibitors.

## Competing interests

The authors declare that they have no competing interests.

## Authors’ contributions

LMS read all studies reviewed herein and drafted the document. FCD, MBV, MKD, and SJD conducted literature searches and edited the document. All authors read and approved the final manuscript.
